# Association between albumin-corrected anion gap and 28-day mortality in critically ill patients with pneumonia in the intensive care unit: evidence from the medical information mart for intensive care IV database

**DOI:** 10.1186/s12890-026-04207-0

**Published:** 2026-02-25

**Authors:** Jilun Hao, Yan Yang, Huaidong Fu, Jialong Zhang

**Affiliations:** 1 Department of Critical Care Medicine, Wuxi Huishan Traditional Chinese Medicine Hospital, Wuxi, Jiangsu 214000 China; 2 Department of Critical Care Medicine, The First people’s Hospital of Yancheng, The Yancheng Clinical College of Xuzhou Medical University, Yancheng, Jiangsu 224008 China

**Keywords:** Albumin-corrected anion gap, Pneumonia, 28-day mortality, Medical Information Mart for Intensive Care IV

## Abstract

**Background:**

The albumin-corrected anion gap (ACAG) has been shown to be associated with prognosis in critically ill patients. However, few studies have investigated the association between ACAG and 28-day mortality in critically ill patients with pneumonia. We hypothesized that the initial ACAG level could predict 28-day mortality risk in critically ill patients with pneumonia.

**Methods:**

This retrospective observational cohort study used the Medical Information Mart for Intensive Care-IV database. Demographic characteristics, vital signs, laboratory parameters, comorbidities, and severity scores were extracted from the first 24 h of intensive care unit admission. Kaplan–Meier curves with log-rank tests were generated to visualize unadjusted survival differences according to ACAG tertiles. Multivariable Cox proportional hazards regression models employing restricted cubic splines (four knots) were used to evaluate the nonlinear association between serum ACAG concentration and 28-day mortality. Effect modification was assessed through interaction and stratified analyses across prespecified subgroups (age, sex, and comorbidity burden).

**Results:**

Among 4,630 intensive care unit patients with pneumonia, 1,364 (29.5%) died by day 28. Each 1-mmol/L increment in ACAG independently predicted high mortality (adjusted hazard ratio [*HR*] 1.02, 95% confidence interval [*CI*] 1.01–1.03; *p* < 0.001), exhibiting dose dependency (10-mmol/L increase: *HR* 1.24, 95% *CI* 1.11–1.38). The highest ACAG tertile demonstrated a 19% greater mortality risk versus the lowest (*HR* 1.19, 95% *CI* 1.01–1.39), whereas the intermediate tertile showed a non-significant association (*HR* 1.07, 95% *CI* 0.92–1.24). Restricted cubic splines confirmed a monotonic dose–response relation (non-linearity, *p* = 0.423), with Kaplan–Meier curves revealing progressively worse survival across the ascending tertiles (log-rank *p* < 0.0001). Subgroup analyses indicated consistent effects across strata, with sepsis status as the sole significant modifier (interaction *p* = 0.025).

**Conclusion:**

In critically ill pneumonia patients, ACAG may be a practical prognostic biomarker, and prospective studies with external validation are recommended.

## Background

Pneumonia remains a significant global health concern, particularly among critically ill patients requiring intensive care unit (ICU) admission [1, 2]. Pneumonia is responsible for a substantial healthcare burden, with estimated costs reaching 10 billion U.S. dollars annually and accounting for more than 2.5 million deaths worldwide each year [3, 4]. According to the Global Burden of Disease Study, pneumonia is the leading infectious disease resulting in hospitalization and death, contributing to considerable healthcare resource utilization and socioeconomic burden [5].

In recent years, the albumin-corrected anion gap (ACAG) has shown potential value as a biomarker that integrates metabolic status and nutritional indicators in prognostic assessment for critical illness. Elevated ACAG is independently associated with mortality in critical illnesses, such as sepsis [6], acute kidney injury [7],congestive heart failure [8],acute respiratory failure [9],and chronic obstructive pulmonary disease [10], and may affect prognosis by reflecting pathological and physiological processes, such as hidden acidosis, hypoalbuminemia, and electrolyte imbalances [11, 12]. However, the relation between ACAG and 28-day mortality in a specific population of critically ill patients with pneumonia has not been systematically elucidated, and its predictive efficacy and underlying mechanisms require high-quality evidence.

Therefore, we conducted a large retrospective cohort study using the Medical Information Mart for Intensive Care (MIMIC)-IV database to explore the association between ACAG and 28-day mortality in this patient group. We hypothesized that high ACAG levels would be associated with an increased risk of 28-day mortality in critically ill patients with pneumonia and that a simple ACAG measurement might serve as a useful laboratory predictor of prognosis in these individuals.

## Methods

### Data source

This study used data from the MIMIC-IV, version 3.1 database [13, 14]. The database was funded by the National Institutes of Health, the Massachusetts Institute of Technology, and Beth Israel Deaconess Medical Center. It was collaboratively developed by emergency physicians, critical care physicians, and computer science experts. The database includes patients admitted to the ICU at Beth Israel Deaconess Medical Center from 2008 to 2019. One of the authors, Jilun Hao, completed the Collaborative Institutional Training Initiative examination, received authorization to access the MIMIC-IV database, and obtained certification (ID: 55,518,033). Patient consent and ethical approval were not deemed necessary for this study, and all patient identifiers were appropriately removed.

### Study participants

All consecutive adult ICU patients (aged > 18 years) diagnosed with pneumonia using the International Classification of Diseases, ICD-9 (codes 480–486) and ICD-10 (codes J12–J18), were selected for the present study [15–17]. Only the first hospitalization and first ICU admission records were included in the analysis. Patients without albumin or serum AG measurements within the first 24 h after ICU admission were excluded. Consequently, the analyzed cohort comprised 4,630 patients.

### Variable extraction

Data were extracted from the MIMIC-IV v3.1 relational database using SQL queries executed in PostgreSQL 10.13. We extracted data on demographic parameters (age, sex, and race), vital signs, laboratory tests, comorbidities, mortality, length of ICU stay, and scoring systems from the first 24 h after ICU admission. Vital signs, including heart rate, mean arterial pressure, respiratory rate, and temperature, were expressed as mean values. The following laboratory parameters were also extracted: AG, white blood cell (WBC) count, hematocrit, hemoglobin, platelet count, creatinine, blood urea nitrogen, albumin, and glucose. Comorbidities included congestive heart failure, renal disease, cerebrovascular disease, peptic ulcer disease, malignancy, and sepsis. We also calculated the Charlson Comorbidity Index (CCI) and Simplified Acute Physiology Score II (SAPSⅡ) for each patient. Sepsis was determined based on the Sequential Organ Failure Assessment score (SOFA) [18], which incorporates six systems: respiratory, cardiovascular, hepatic, coagulation, renal, and neurological. The endpoint of our study was 28-day mortality. The anion gap (AG) was calculated as: AG (mmol/L) = (sodium + potassium) − (chloride + bicarbonate). The ACAG value was calculated using the formula: ACAG (mmol/L) = AG + [44 – albumin (g/L)] × 0.25 [19]. All laboratory variables were collected on the first day of ICU admission, and scores were based on indicators within the first 24 h of admission. Missing AG (*n* = 3) and albumin (*n* = 4,175) data were excluded, while missing SOFA scores (*n* = 12) were replaced with mean values.

### Statistical analysis

Patients were stratified into three groups based on tertiles of ACAG distribution. Continuous variables are presented as mean ± standard deviation or median (interquartile range), as appropriate. Categorical variables are expressed as counts and percentages. Intergroup comparisons for continuous variables were performed using the two-sample *t*-test or Mann–Whitney U test, whereas categorical variables were compared using the chi-squared test.

Multivariable Cox proportional hazards regression models were used to assess the association between ACAG tertiles and 28-day mortality risk by calculating hazard ratios (*HR*s) with 95% confidence intervals (*CI*s). The lowest tertile of ACAG served as the reference group. Three sequential adjustment models were constructed:

- Model I: Adjusted for age, sex, and race.
- Model II: Adjusted for Model I variables plus vital signs (heart rate, respiratory rate, mean arterial pressure, and temperature).
- Model III: Adjusted for Model II variables plus comorbidities (congestive heart failure, cerebrovascular disease, peptic ulcer disease, renal disease, malignancy, and sepsis), laboratory parameters (platelet count, blood urea nitrogen, WBC count, creatinine, hematocrit, hemoglobin, and glucose), and Charlson Comorbidity Index.

Subgroup analyses were conducted to evaluate the potential effect modification of the ACAG-mortality association across patient subgroups.

All analyses were performed using *R* Statistical Software (Version 4.2.2 http://www.R-project.org, The *R* Foundation) and Free Statistics analysis platform (Version 2.3). Statistical significance was defined as a two-sided *p*-value < 0.05.

## Results

### Participant characteristics

**Fig. 1:**
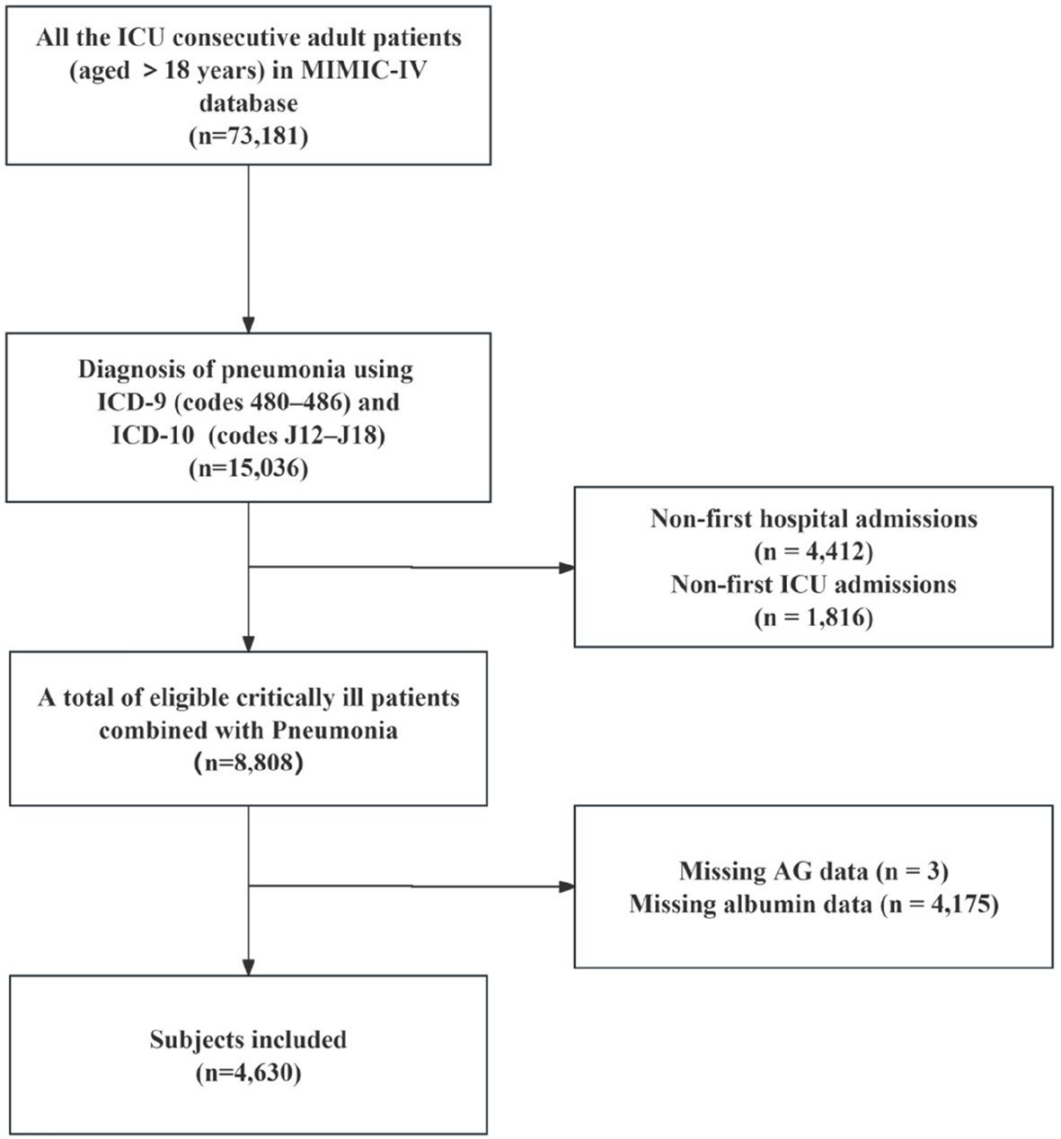
Complete data regarding the inclusion and exclusion processes. ICU intensive care unit, MIMIC-IV Medical Information Mart for Intensive Care IV, AG Anion Gap

The MIMIC-IV database contained 8,808 critically ill patients with pneumonia. After excluding 4,178 patients because of missing data (see Fig. 1 for details), 4,630 patients were included in the final analysis, with 56.9% men and 43.1% women, and a median age of 65.5 years. Among these patients, the 28-day mortality was 29.5%. Baseline characteristics of the ACAG tertiles are presented in Table 1. The baseline feature comparison between the final analysis queue (*n* = 4630) and patients excluded due to missing data (*n* = 4178) is shown in Supplementary Table 1.

**Table 1:** Baseline Characteristics of Participants According to ACAG

Variables	Total	ACAG(mmol/L)			
		T1 (≤ 18)	T2 (18–21.75)	T3 (≥ 21.75)	*p*
Patients (*N*)	4630	1502	1554	1574	
Gender, *N* (%)					0.042
Female	1995 (43.1)	650 (43.3)	703 (45.2)	642 (40.8)	
Male	2635 (56.9)	852 (56.7)	851 (54.8)	932 (59.2)	
Race, *N* (%)					< 0.001
White	2721 (58.8)	882 (58.7)	951 (61.2)	888 (56.4)	
Black	487 (10.5)	128 (8.5)	144 (9.3)	215 (13.7)	
Others	1422 (30.7)	492 (32.8)	459 (29.5)	471 (29.9)	
Age(years)	65.5 ± 16.5	65.3 ± 16.7	66.0 ± 16.6	65.3 ± 16.4	0.35
Vital signs					
HR (bmp)	89.6 ± 17.5	85.0 ± 16.2	89.8 ± 16.8	93.9 ± 18.1	< 0.001
MBP (mmHg)	78.1 ± 10.9	79.7 ± 10.5	77.8 ± 10.6	76.7 ± 11.3	< 0.001
RR (bmp)	21.7 ± 4.6	20.9 ± 4.4	21.7 ± 4.5	22.5 ± 4.7	< 0.001
Temperature (℃)	36.9 ± 0.6	37.0 ± 0.5	37.0 ± 0.6	36.9 ± 0.7	< 0.001
Comorbidity, *N* (%)					
CHF	1526 (33.0)	442 (29.4)	542 (34.9)	542 (34.4)	0.002
Cerebrovascular disease	644 (13.9)	238 (15.8)	206 (13.3)	200 (12.7)	0.028
Peptic ulcer disease	154 (3.3)	38 (2.5)	53 (3.4)	63 (4)	0.073
Renal disease	1066 (23.0)	222 (14.8)	359 (23.1)	485 (30.8)	< 0.001
vSepsis	3677 (79.4)	1067 (71)	1253 (80.6)	1357 (86.2)	< 0.001
Laboratory parameters					
Glucose (mg/dL)	133.0(110.0,172.5)	126.8(108.3, 154.0)	132.7(109.5, 167.3)	145.1 (114.0, 194.5)	< 0.001
WBC (10^9^/L)	13.5 (9.3, 18.8)	11.6 (8.2, 16.0)	13.5 (9.6, 18.4)	15.6 (10.6, 22.4)	< 0.001
Platelets (10^9^/L)	191.9 ± 117.1	199.7 ± 112.0	201.5 ± 122.8	175.1 ± 114.4	< 0.001
Hemoglobin (g/dL)	10.1 ± 2.4	10.5 ± 2.3	10.1 ± 2.3	9.6 ± 2.4	< 0.001
Hematocrit (%)	30.9 ± 7.2	32.1 ± 6.9	30.9 ± 7.1	29.6 ± 7.3	< 0.001
BUN (mg/dL)	27.0(17.0,45.0)	21.0(14.0,31.0)	26.0(17.0, 41.0)	40.0 (24.0, 65.0)	< 0.001
Creatinine (mg/dL)	1.2 (0.8, 2.1)	0.9 (0.7, 1.3)	1.2 (0.8, 1.8)	2.0 (1.2, 3.7)	< 0.001
Albumin (g/dL)	3.1 ± 0.7	3.3 ± 0.6	3.0 ± 0.6	2.9 ± 0.7	< 0.001
Specimen, *N* (%)					0.003
Sputum culture	1547 (33.4)	496 (33)	499 (32.1)	552 (35.1)	
Blood culture	389 (8.4)	101 (6.7)	132 (8.5)	156 (9.9)	
Scoring systems					
CCI	6.2 ± 3.1	5.7 ± 3.0	6.2 ± 3.1	6.6 ± 3.1	< 0.001
SOFA	6.0 ± 3.8	4.5 ± 3.1	5.6 ± 3.5	7.9 ± 4.1	< 0.001
SAPSⅡ	41.6 ± 15.2	36.2 ± 13.0	40.0 ± 13.5	48.3 ± 16.3	< 0.001
Outcomes					
28- day mortality, N (%)	1364 (29.5)	325 (21.6)	420 (27)	619 (39.3)	< 0.001

*Abbreviations*: *T* tertile, *ICU* Intensive Care Unit, *HR* heart rate, *bpm* beats per minute, *MBP* mean blood pressure, *RR* respiratory rate, *CHF* congestive heart failure, *WBC* white blood cells, *BUN* blood urea nitrogen, *CCI* Charlson Comorbidity Index, *SOFA* Sequential Organ Failure Assessment score, *SAPSII* Simplified Acute Physiology Score II

### Association between ACAG and 28-day mortality

In univariate Cox proportional hazards analysis, the risk of 28-day mortality increased significantly with rising ACAG levels (*HR* = 1.06, 95% *CI*: 1.05–1.07; *p* < 0.001; Table 2). When analyzed by ACAG tertiles, mortality risks relative to the lowest tertile (T1) demonstrated a dose–response relation: T2 (*HR* = 1.30, 95% *CI*: 1.13–1.50) and T3 (*HR* = 2.11, 95% *CI*: 1.85–2.42). The same univariate models revealed an elevated mortality risk with advancing age, higher heart rate, increased respiratory rate, elevated WBC count, increased blood urea nitrogen, elevated creatinine levels. Higher scores across all illness severity scoring systems (CCI, SOFA, SAPS II) were strongly predictive of mortality (all *p* < 0.001). All examined comorbidities, except peptic ulcer disease, were positively associated with 28-day mortality. Conversely, temperature, mean arterial pressure, hematocrit, hemoglobin, and platelet count were inversely correlated with mortality risk.

**Table 2:** Association Between ACAG and Covariates with 28 day Mortality Risk

Item	*HR*(95%*CI*)	*P*(LR-test)
ACAG (mmol/L)	1.06 (1.05,1.07)	< 0.001
ACAG group(mmol/L)		< 0.001
T1	1.00 (Ref)	
T2	1.30 (1.13,1.50)	
T3	2.11 (1.85,2.42)	
Gender		
Female	1.00 (Ref)	
Male	0.92 (0.83,1.03)	0.142
Race		< 0.001
White	1.00 (Ref)	
Black	0.95 (0.79,1.14)	
Others	1.24 (1.10,1.39)	
Age(years)	1.02 (1.02,1.03)	< 0.001
Age(years)		
Age < 65	1.00 (Ref)	
Age ≥ 65	1.90 (1.69,2.13)	< 0.001
Scoring systems		
CCI	1.15 (1.13,1.17)	< 0.001
SOFA	1.12 (1.11,1.14)	< 0.001
SAPSⅡ	1.04 (1.04,1.05)	< 0.001
Vital sings		
HR (bpm)	1.01 (1.01,1.01)	< 0.001
MBP (mmHg)	0.97 (0.97,0.98)	< 0.001
RR (bpm)	1.04 (1.02,1.05)	< 0.001
Temperature (℃)	0.68 (0.62,0.73)	< 0.001
Comorbidity		
NO	1.00 (Ref)	
CHF	1.11 (1.00,1.24)	0.061
Cerebrovascular disease	1.33 (1.15,1.53)	< 0.001
Peptic ulcer disease	0.94 (0.69,1.27)	0.668
Renal disease	1.27 (1.12,1.43)	< 0.001
Sepsis	1.40 (1.21,1.62)	< 0.001
Laboratory parameters		
Glucose (mg/dL)	1.00 (0.99,1.00)	0.674
WBC (109/L)	1.00 (1.00,1.01)	< 0.001
Platelets (10^9^/L)	0.99 (0.98,0.99)	< 0.001
Hemoglobin (g/dL)	0.92 (0.90,0.94)	< 0.001
Hematocrit (%)	0.98 (0.97,0.99)	< 0.001
BUN (mg/dL)	1.01 (1.01,1.01)	< 0.001
Creatinine (mg/dL)	1.06 (1.03,1.08)	< 0.001
Albumin, (g/dL)	0.58 (0.54,0.63)	< 0.001

*Abbreviations*: *HR* hazard ratio, *CI* confidence interval, *Ref* reference, *T* tertile, *CCI* Charlson Comorbidity Index, *bpm* beats per minute, *HR* heart rate, *MBP* mean blood pressure, *RR* respiratory rate, *CHF* congestive heart failure, *WBC* white blood cells, *BUN* blood urea nitrogen, *SOFA* Sequential Organ Failure Assessment score, *SAPSII* Simplified Acute Physiology Score II

### Multivariable analysis of ACAG and mortality risk

In fully adjusted multivariable Cox proportional hazards models (Model III), each 1 mmol/L increase in ACAG remained significantly associated with 28-day mortality (*HR* = 1.02, 95% *CI*: 1.01–1.03; *p* < 0.001;Table 3). This corresponds to a 24% higher mortality risk per 10 mmol/L increment (adjusted *HR* = 1.24, 95% *CI*: 1.11–1.38; *p* < 0.001;Table 3).

**Table 3:** Association Between ACAG and 28 day Mortality Risk in Different Models

Variable	n.total	N (%)	Crude Model		Model-Ⅰ		Model-Ⅱ		Model-Ⅲ	
			***HR* (95%CI)**	***P* value**	***HR* (95%CI)**	***P* value**	***HR* (95%CI)**	***P* value**	***HR* (95%CI)**	***P* value**
ACAG(mmol/L)	4630	1364(29.5)	1.06(1.05 ~ 1.07)	< 0.001	1.07(1.06 ~ 1.08)	< 0.001	1.05 (1.04 ~ 1.06)	< 0.001	1.02 (1.01 ~ 1.03)	< 0.001
ACAG(10 mmol/L)	4630	1364(29.5)	1.81(1.67 ~ 1.96)	< 0.001	1.91(1.77 ~ 2.07)	< 0.001	1.63 (1.5 ~ 1.78)	< 0.001	1.24 (1.11 ~ 1.38)	< 0.001
ACAG.tertiles(mmol/L)										
T1(≤ 18)	1502	325 (21.6)	1.00 (Ref)		1.00 (Ref)		1.00 (Ref)		1.00 (Ref)	
T2(18–21.75)	1554	420 (27)	1.3 (1.13 ~ 1.5)	< 0.001	1.3 (1.12 ~ 1.5)	< 0.001	1.15 (0.99 ~ 1.33)	0.06	1.07 (0.92 ~ 1.24)	0.365
T3(≥ 21.75)	1574	619 (39.3)	2.11 (1.85 ~ 2.42)	< 0.001	2.19 (1.91 ~ 2.51)	< 0.001	1.71 (1.49 ~ 1.97)	< 0.001	1.19 (1.01 ~ 1.39)	0.033
Trend.test				< 0.001		< 0.001		< 0.001		0.031

Adjustment Sets:Crude Model: Unadjusted.Model I: Age, sex, race.Model II: Model I variables + vital signs (heart rate, respiratory rate, mean arterial pressure, temperature).Model III: Model II variables + comorbidities (congestive heart failure, cerebrovascular disease, peptic ulcer disease, renal disease, malignancy, sepsis), laboratory parameters (platelet count, BUN, WBC, creatinine, haematocrit, haemoglobin, glucose), and Charlson Comorbidity Index,SAPSⅡ,SOFA

*Abbreviations*: *HR* hazard ratio, *CI* confidence interval, *Ref* reference, *T* tertile, *CCI* Charlson Comorbidity Index, *bpm* beats per minute, *HR* heart rate, *MBP* mean blood pressure, *RR* respiratory rate, *CHF* congestive heart failure, *WBC* white blood cells, *BUN* blood urea nitrogen, *SOFA* Sequential Organ Failure Assessment score, *SAPSII* Simplified Acute Physiology Score II

When analyzed by tertiles, compared with the reference group (T1: ≤ 18 mmol/L), the fully adjusted *HR* for T2 (18–21.75 mmol/L) was 1.07 (95% *CI*: 0.92–1.24; *p* = 0.365) and for T3 (≥ 21.75 mmol/L) was 1.19 (95% *CI*: 1.01–1.39; *p* = 0.033). Significant dose–response relations persisted across progressively adjusted models (*p* for trend < 0.05 for all models;Table 3).

### Restricted cubic spline analysis and Kaplan–Meier survival curves

**Fig. 2:**
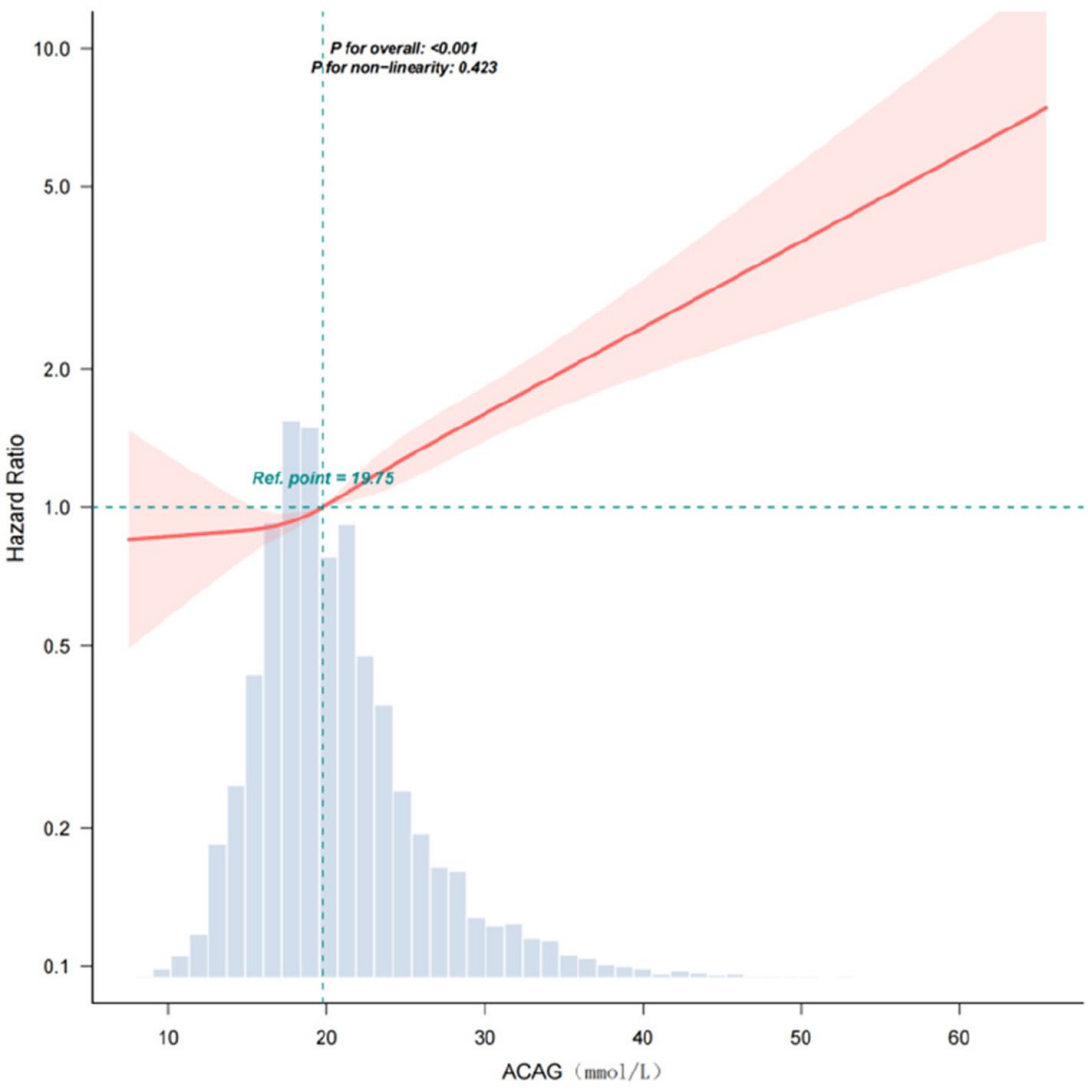
Correlation of ACAG with the risk of 28-day mortality. Abbreviations: ACAG albumin corrected anion gap

Restricted cubic spline analysis demonstrated a significant overall association between ACAG and 28-day mortality (*p* < 0.001 for the overall effect), and no evidence of nonlinearity was observed (*p* for nonlinearity = 0.423), confirming a linear dose–response relation across the ACAG continuum (Fig. 2). A reference point (*HR* = 1.0) was established at 19.75 mmol/L, with progressively increasing mortality risk observed at higher ACAG concentrations.

**Fig. 3:**
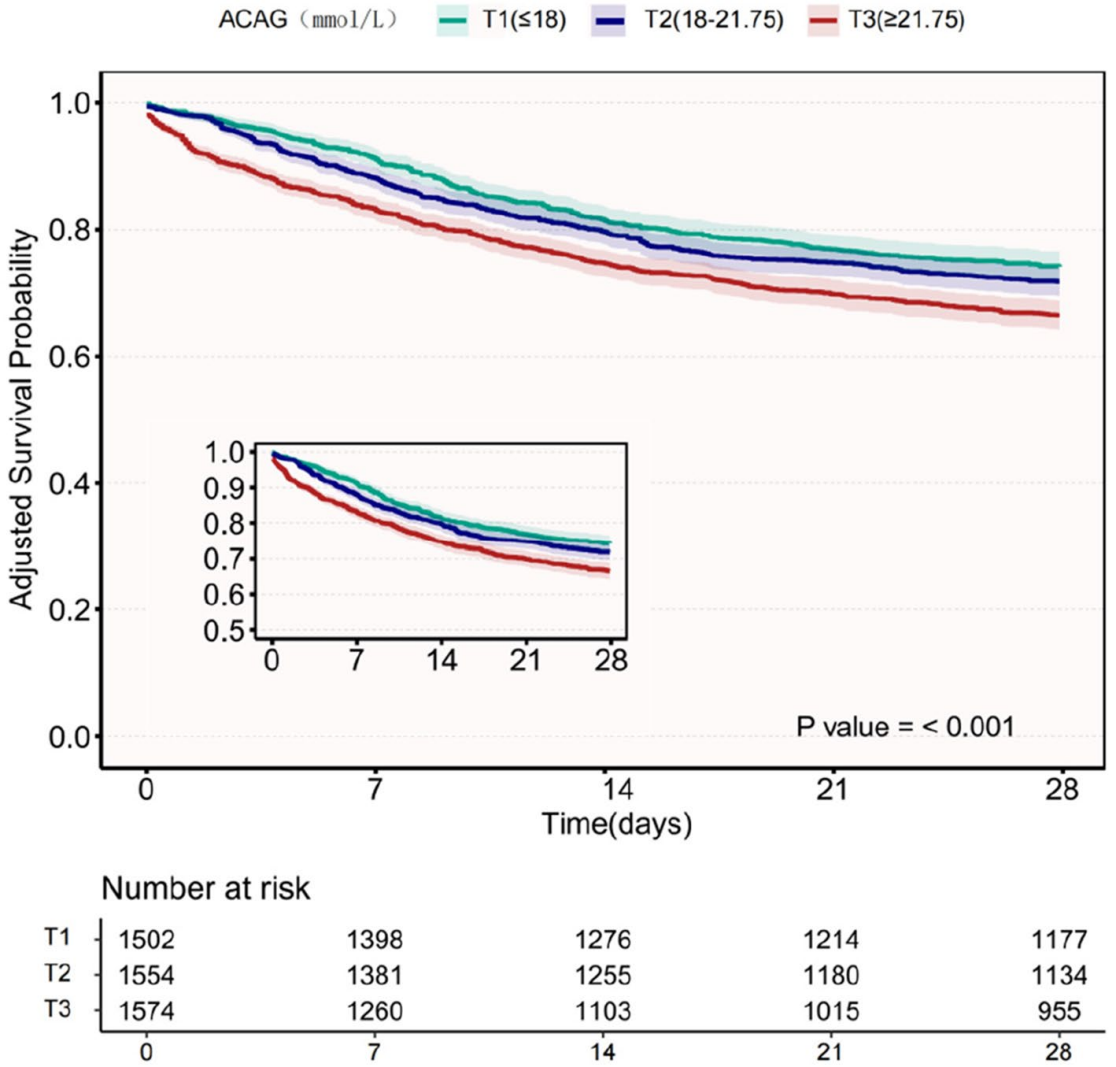
Kaplan–Meier curves of 28-day cumulative survival rates. Adjustment Sets: Age, sex, race, vital signs (heart rate, respiratory rate, mean arterial pressure, temperature), comorbidities (congestive heart failure, cerebrovascular disease, peptic ulcer disease, renal disease, malignancy, sepsis), laboratory parameters (platelet count, BUN, WBC, creatinine, haematocrit, haemoglobin, glucose), Charlson Comorbidity Index. Abbreviations: ACAG albumin corrected anion gap; T, tertile; BUN, blood urea nitrogen; WBC, white blood cells

Kaplan–Meier curves stratified by ACAG tertiles demonstrated significantly divergent 28-day survival rates (log-rank *p* < 0.001). From T1 (≤ 18 mmol/L, *n* = 1,502) to T3 (≥ 21.75 mmol/L, *n* = 1,574), adjusted survival probabilities decreased progressively (78.4% vs. 60.7%; *p* for trend < 0.001), demonstrating a dose-dependent reduction in survival with increasing ACAG levels (Fig. 3).

### Subgroup analysis

**Fig. 4:**
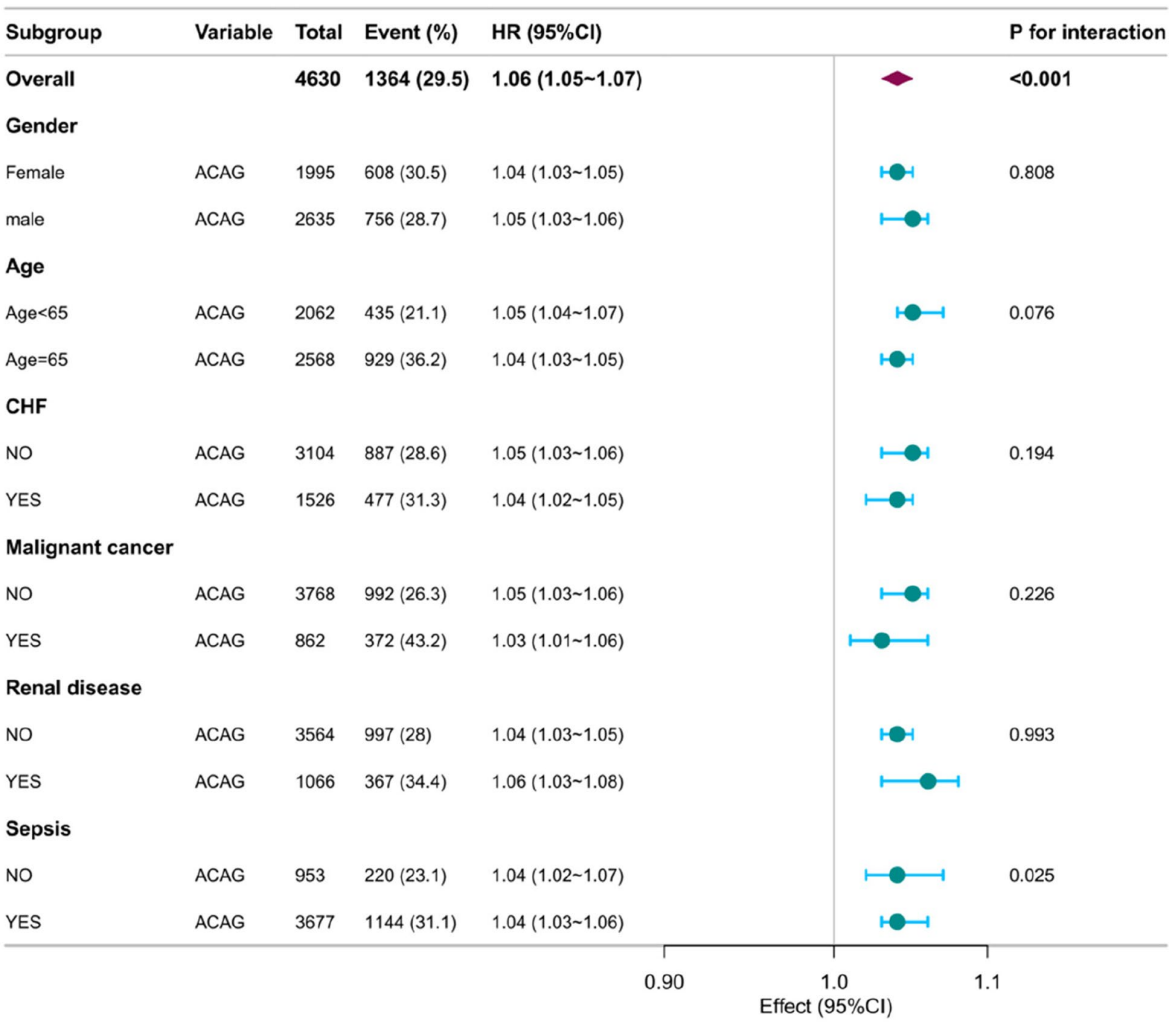
The association between ACAG and 28-day mortality in different subgroups. Abbreviations: ACAG albumin corrected anion gap, *HR* hazard ratio, *CI* confidence interval;CHF, congestive heart failure

To further examine the robustness of the relation between ACAG levels and 28-day mortality, we conducted a subgroup analysis based on sex, age, and comorbidities, as shown in the forest plot in Fig. 4. The results indicated that the association between ACAG and mortality was consistent across patients of different sexes, ages, and comorbidity profiles. Interaction analysis revealed that sepsis modified the association between ACAG and mortality (*p* = 0.025).

## Discussion

In this retrospective cohort study of 4,630 critically ill patients, an elevated ACAG independently predicted increased 28-day mortality in a dose-dependent manner (*p* for trend < 0.001). This association persisted after comprehensive adjustment for demographics, vital signs, key laboratory parameters, and comorbidity burden (Charlson Comorbidity Index) and demonstrated consistent directionality across predefined clinical subgroups.

Pneumonia remains a leading cause of morbidity and mortality worldwide and is a frequent indication for ICU admission. Global estimates consistently place lower respiratory tract infections among the top contributors to infectious disease burden and in-hospital death, particularly in older adults and patients with comorbidities [4]. Severe community- and hospital-acquired pneumonia frequently progress to sepsis, respiratory failure, and multiorgan dysfunction, thereby driving short-term mortality in the critically ill [18, 20]. Therefore, early and reliable risk stratification of ICU patients with pneumonia is essential to guide resuscitation, antimicrobial strategies, and escalation of organ support therapies.

Recent research has renewed interest in simple, routinely available laboratory indices as prognostic biomarkers for critical illness. The serum AG, historically used in acid–base evaluation, reflects unmeasured anions and increases with the accumulation of organic acids (including lactate), renal retention of anions, and ingestion of toxins [11, 21, 22]. Albumin is a major unmeasured anion; hypoalbuminemia lowers AG and can mask the underlying acid load. Therefore, correction of AG for serum albumin concentration (ACAG) has been advocated to improve diagnostic and prognostic accuracy in diverse clinical contexts [23, 24]. Compared with AG, ACAG more accurately identifies occult acidosis [25], is more conducive to the diagnosis of lactic acidosis, and more reliably characterizes acid–base disturbances [6, 26].Several retrospective cohort studies in heterogeneous ICU populations and specific disease states (including sepsis and cardiogenic shock) have reported associations between elevated ACAG levels and short-term mortality, suggesting incremental prognostic value beyond conventional severity scores [27, 28]. However, data addressing ACAG in critically ill patients with pneumonia remain limited.

Our findings extend prior work by quantifying the prognostic utility of ACAG in a large, heterogeneous ICU population and demonstrating an approximately linear relation between ACAG and short-term mortality. Mechanistically, ACAG integrates unmeasured anions and corrects for hypoalbuminemia, which frequently masks an elevated AG. Thus, a higher ACAG may reflect more severe metabolic acidosis, impaired renal clearance of acid, or broader metabolic derangements linked to worse outcomes. Pneumonia, particularly under severe hypoxia, can cause cellular oxygen deprivation and the production of various metabolic byproducts, triggering metabolic acidosis and increasing AG. Hypoalbuminemia in ICU patients with pneumonia frequently leads to an underestimated AG. Correcting AG for albumin unmasks the acid burden and integrates two pathophysiologic signals—acid accumulation and protein depletion—that together reflect disease severity and physiologic reserve. Moreover, ACAG may capture contributions from renal dysfunction and the accumulation of unmeasured anions, conditions prevalent in severe pneumonia and associated with higher mortality.

Haessler et al. reported low diagnostic yields in severe community-acquired pneumonia (sCAP), with positive blood cultures in only 10% and positive respiratory cultures (including sputum) in 34.2% of patients [29]. Our findings confirmed these low yields: blood cultures yielded pathogens in 8.4% of cases, while sputum cultures provided diagnostically useful results in 33.4% of patients, respectively. The pathophysiology of pneumonia complicated by sepsis, involving lactate production from hypoperfusion, mitochondrial dysfunction, and hypoalbuminemia (both of which lower AG and predict poor outcomes) [30, 31], aligns with our subgroup analysis finding of a significant interaction between sepsis and ACAG in predicting 28-day mortality.

### Limitations

First, this was an observational study that could not prove causality. Residual confounding and selection bias were possible despite multivariate adjustment. The single-center nature or use of a specific database may limit generalizability; therefore, external validation is needed. Additionally, the reliance solely on International Classification of Diseases (ICD) codes to identify pneumonia cases introduces potential limitations. ICD codes, primarily designed for administrative purposes, may not fully capture clinical nuances and are susceptible to misclassification, potentially biasing the estimated prevalence or associations observed. The exclusion of 4,178 potentially eligible patients (47.4% of pneumonia-identified cohort) due to missing laboratory parameters may introduce selection bias. Serum albumin measurements were unavailable for 4,175 patients (47.4%), limiting comprehensive nutritional status assessment. Mean substitution for missing SOFA scores may flatten severity gradients in organ dysfunction trajectories, though this effect remains minimal at population level given low missingness (0.14%). We used the admission ACAG and did not analyze dynamic changes over time. Some subgroup analyses may also have had limited statistical power. Finally, detailed parameters of fluid resuscitation strategies (e.g., cumulative volume, fluid type, administration timing) in septic patients were not captured. The absence of these dynamic variables in our adjustment models precludes assessment of their confounding effects on outcomes–a limitation meriting investigation in future studies incorporating real-time resuscitation data.

### Clinical implications

ACAG is readily calculable from routine laboratory tests and may serve as an inexpensive adjunct to established risk scores (including PSI, CURB-65, and Sequential Organ Failure Assessment) to identify high-risk patients with pneumonia who might benefit from earlier aggressive management or enrollment in interventional trials. Future work should (1) externally validate ACAG as a prognostic biomarker across diverse ICU populations, (2) evaluate whether serial ACAG trajectories improve discrimination compared with single measurements, (3) explore the incorporation of ACAG into composite risk algorithms, and (4) perform mechanistic studies to delineate the predominant contributors to ACAG elevation in pneumonia (lactate, renal retention, and inflammatory mediators) and assess whether interventions that modify ACAG trajectories affect outcomes.

## Conclusions

In critically ill pneumonia patients, ACAG independently predicted 28-day mortality with monotonic dose–response relationship. While significant associations were confined to extreme ACAG tertiles, ACAG may be a practical prognostic biomarker, and prospective studies with external validation are recommended.

## Data Availability

The data that support the findings of this study are available from the corresponding author upon reasonable request.

## Abbreviations

ICU: Intensive care unit
ACAG: Albumin-corrected anion gap
MIMIC: Medical Information Mart for Intensive Care
WBC: White blood cell
*HR*s: Hazard ratios
*Ci*s: Confidence intervals
